# The role of cellular prion protein in lipid metabolism in the liver

**DOI:** 10.1080/19336896.2020.1729074

**Published:** 2020-03-05

**Authors:** Amandeep Singh Arora, Saima Zafar, Umair Latif, Franc Llorens, Mihm Sabine, Prateek Kumar, Waqas Tahir, Katrin Thüne, Mohsin Shafiq, Matthias Schmitz, Inga Zerr

**Affiliations:** a Department of Neurology, Clinical Dementia Center, University Medical Center Göttingen and German Center for Neurodegenerative Diseases (DZNE), Göttingen, Germany; b Departments of Gastroenterology Endocrinology, University Medical Center Göttingen, Göttingen, Germany; c Network Center for Biomedical Research in Neurodegenerative Diseases, (CIBERNED), Institute Carlos III, Hospitalet De Llobregat, Spain

**Keywords:** PrPC, liver, proteomics, ingenuity pathway analysis, non-alcoholic fatty liver disease

## Abstract

Cellular prion protein (PrPC) is a plasma membrane glycophosphatidylinositol-anchored protein and it is involved in multiple functions, including neuroprotection and oxidative stress. So far, most of the PrPC functional research is done in neuronal tissue or cell lines; the role of PrPC in non-neuronal tissues such as liver is only poorly understood. To characterize the role of PrPC in the liver, a proteomics approach was applied in the liver tissue of PrPC knockout mice. The proteome analysis and biochemical validations showed an excessive fat accumulation in the liver of PrPC knockout mice with a change in mRNA expression of genes linked to lipid metabolism. In addition, the higher Bax to Bcl2 ratio, up-regulation of tgfb1 mRNA expression in PrPC knockout mice liver, further showed the evidences of metabolic disease. Over-expression of PrPC in fatty acid-treated AML12 hepatic cell line caused a reduction in excessive intracellular fat accumulation; shows association of PrPC levels and lipid metabolism. Therefore, based on observation of excessive fat globules in the liver of ageing PrPC knockout mice and the reduction of fat accumulation in AML12 cell line with PrPC over-expression, the role of PrPC in lipid metabolism is described.

## 1. Introduction

PrPC (prion protein *c*ellular) is a plasma membrane glycoprotein and its misfolded, proteinase K resistant isoform called PrPSc (prion protein *Sc*rapie) is known as a causative agent of transmissible spongiform encephalopathies (TSE) in humans and animals [1]. PrPC is widely expressed in the body with the highest abundance in neuronal tissues and involved in multiple functions [1]. Due to its conserved gene sequence in different species, PrPC is supposed to have fundamental functions. The expression of PrPC in the peripheral tissues is comparatively low and far from being understood. Though earlier reports in 1992–1993 showed that autopsy of patients from Cruetzfeldet-Jakob disease is associated with fatty liver [2,3], no further study has been followed-up to understand and characterize the exact molecular function of PrPC in the liver. Though a recent study demonstrated that PrPC regulates the lipids level in adipose tissue by negatively regulating autophagy pathway [4]. In addition, it was earlier reported that inhibiting the expression of PrPC in glial and non-glial cancer cells induces cell death by activating autophagy [5]. However, the role of PrPC in fatty liver or lipid metabolism in the liver is not yet known.

Further, a few studies have reported that increased PrPC expression in liver is associated with liver fibrosis in Chronic Viral Hepatitis [6] and the proliferation of hepatic stellate cells (HSCs) [7]. Therefore, the aim of the current study is to elucidate the detailed molecular and cellular mechanism of PrPC in the liver.

It is known that protein expression of PrPC in the liver is very low [8–11], and we had earlier reported a significant age-dependent up-regulation of PrPC expression in the liver [12]. In addition, the expression of PrPC is significantly higher in females as compared to males. Based on our previous study that the expression of PrPC increases in the liver of ageing mice and human, we intended to understand and characterize the hepatic function of PrPC with proteomics approach in the liver tissue specimens from PrPC knockout *Zürich I* and wild type mice of both sexes at different ages (3, 9 and 14 month-old). We used two-dimensional gel electrophoresis-based proteomics approach in all age groups and the gel-free quantitative proteomics in the 14 months only.

Proteomics results indicated that the liver of PrPC knockout mice may have an excessive deposition of fat in 14 months age and phenotype was subsequently validated by Sudan III lipid stain and mRNA levels of genes involved in lipogenesis. Further, *in vitro* experiments validated that the negative regulation of autophagy by PrPC in AML12 hepatocyte cell line regulates the intracellular excessive fat levels.

## 2. Results

### 2.1. 2D gel electrophoresis of PrPC knockout mice liver

Liver samples from 3, 9 and 14 month-old *Zürich I* PrPC knockout and wild type mice of both sexes were subjected to 2D gel electrophoresis-based proteomics approach. In total, 46 gels (17 cm width) from liver tissues of 3, 9 and 14 month-old (PrPC knockout and wild type with 4/3 mice from each group) with well-separated spots were obtained (Details of biological replicates are provided in the supporting information, Table 3S). The images of each gel were subjected to differential spot analysis by Delta2D. The image analyses revealed 3035 protein spots and each spot was identified with multiple sets of proteins. The comparison of PrPC knockout and wild type mice gels revealed 26 differentially regulated spots (Supplementary Figures 1S and 2s) and proteins with the highest score/spectral count are shown in Table 1. However, more than one protein was detected by mass spectrometry in each spot and the detailed list of all proteins detected in each spot is presented in the supporting information (Table 1S, *supporting information*). In total, 8 spots were regulated in the liver of 3 and 14 month-old PrPC knockout male mice as compared to the wild type while no significantly regulated spot was found in the 9 months male group. Eighteen spots were found to be regulated in the liver of 3, 9 and 14 month-old PrPC knockout female mice as compared to the wild type.

**Table 1.** Detailed list of statistically significant (*P*-value ≤ 0.05) dataset from mass spectrometry analysis: List included regulated proteins in the liver of 3, 9 and 14 month-old of PrPC knockout mice as compared to the wild type (No significant-regulated gel spots were found in the liver of 9 month-old male PrPC knockout mice). Each spot containing proteins with the highest spectral count (score) is present in the table. The screening of the raw dataset was done by their unique molecular mass and isoelectric pH (*pI*).

ID	Protein name	Accession number	MW (kDa)	Coverage %	Score	Fold Change	*P-value*	*pI*
	**Male – 3-month-old mice**							
1	Isoamyl acetate-hydrolysing esterase 1 homolog	Q9DB29	27.98	36.90%	24	1.55 ↓	0.018	5.34
2	Serine-threonine kinase receptor-associated protein	Q9Z1Z2	38.44	40.30%	26	1.53 ↓	0.014	4.99
3	Regucalcin	Q64374	33.41	56.90%	155	1.59 ↓	0.031	5.16
4	Not identified					4.65 ↓	0.025	
5	Propionyl-CoA carboxylase alpha chain, mitochondrial	Q91ZA3	79.92	17.70%	17	1.58 ↓	0.04	6.04
	**Male – 14-month-old mice**							
6	U5 small nuclear ribonucleoprotein 200 kDa helicase	Q6P4T2	244.55	12.30%	36	1.77 ↓	0.012	5.73
7	3-mercaptopyruvate sulfurtransferase	Q99J99	33.02	67.30%	68	6.70 ↑	≤0.001	6.12
8	Not identified					1.53 ↓	0.046	
	**Female – 3-month-old mice**							
9	Farnesyl pyrophosphate synthase	Q920E5	40.58	42.80%	105	2.28 ↓	0.02	5.48
10	Annexin A5	P48036	35.75	59.90%	182	2.16 ↓	0.006	4.82
11	Alpha-soluble NSF attachment protein	Q9DB05	33.19	70.5%	146	2.14 ↓	0.013	5.30
12	3-mercaptopyruvate sulfurtransferase	Q99J99	33.02	67.30%	68	3.54 ↑	0.04	6.12
13	Vacuolar protein sorting-associated protein 29	Q9QZ88	20.50	44.50%	39	2.14 ↑	0.006	6.29
14	14-3-3 protein gamma	P61982	28.30	36.0%	21	2.66 ↑	0.017	4.80
15	39 S ribosomal protein L12, mitochondrial	Q9DB15	21.71	55.70%	48	2.07 ↑	0.004	5.35
	**Female – 9-month-old mice**							
16	Histidine ammonia-lyase	P35492	72.26	14.90%	30	2.05 ↓	0.039	5.94
17	Leukocyte elastase inhibitor A	Q9D154	42.58	44.10%	59	2.19 ↓	0.009	5.85
18	3-mercaptopyruvate sulfurtransferase	Q99J99	33.02	67.30%	68	2.53 ↑	0.019	6.12
19	Proteasome subunit beta type-3	Q9R1P1	22.96	55.60%	103	2.28 ↑	0.04	6.13
	**Female – 14-month-old mice**							
20	Not identified					1.82 ↑	0.012	
21	Stress-70 protein, mitochondrial	P38647	73.46	69.50%	507	1.56 ↓	0.029	5.44
22	Actin, cytoplasmic 1	P60710	41.74	37.60%	19	4.96 ↓	0.0004	5.29
23	Leukocyte elastase inhibitor A	Q9D154	42.58	44.10%	59	2.23 ↑	0.012	5.82
24	Amine sulfotransferase	O35403	35.18	41.3%	201	3.01↓	0.036	6.08
25	Not identified					10.94 ↑	0.031	
26	Putative hydrolase RBBP9	O88851	20.91	19.4%	13	5.27 ↓	0.003	5.62

### 2.2. Proteome analysis

Proteins from all age groups and both sexes were combined together in a single list (*i.e*. 62 proteins) for IPA ingenuity (Qiagen), because of the number of proteins in each group (male and female proteome dataset, separately) were less. The analysis revealed four functional networks (Table 2S, *supporting information*); Network 1: Lipid Metabolism, Small Molecule Biochemistry, Vitamin and Mineral Metabolism, Network 2: Cellular Function and Maintenance, Cellular Growth and Proliferation, Cellular Movement. Network 3: Cancer, Cellular Development, Organismal Injury and Abnormalities. Network 4: Metabolic Disease, Carbohydrate Metabolism, Cardiovascular Disease.

The functional networks with diverse forms of functions were obtained and network 1 has the highest score, which pointed out to the regulation of lipid metabolism (Figure 5 and Table 2S). Liver steatosis or non-alcoholic fatty liver disease is a reversible condition in which large globules containing triglycerides are deposited in the hepatocytes. In addition, IPA functional networks also indicated a metabolic syndrome in the liver of PrPC knockout mice as compared to the wild type (Network 4, mentioned above). Therefore, first the amount of triglyceride levels were measured, followed by histopathological examination with H and E (Hematoxylin and Eosin) and Sudan III fat stain.

### 2.3. Triglyceride content (mg/dl) in the liver of PrPC knockout mice

**Figure 1.**
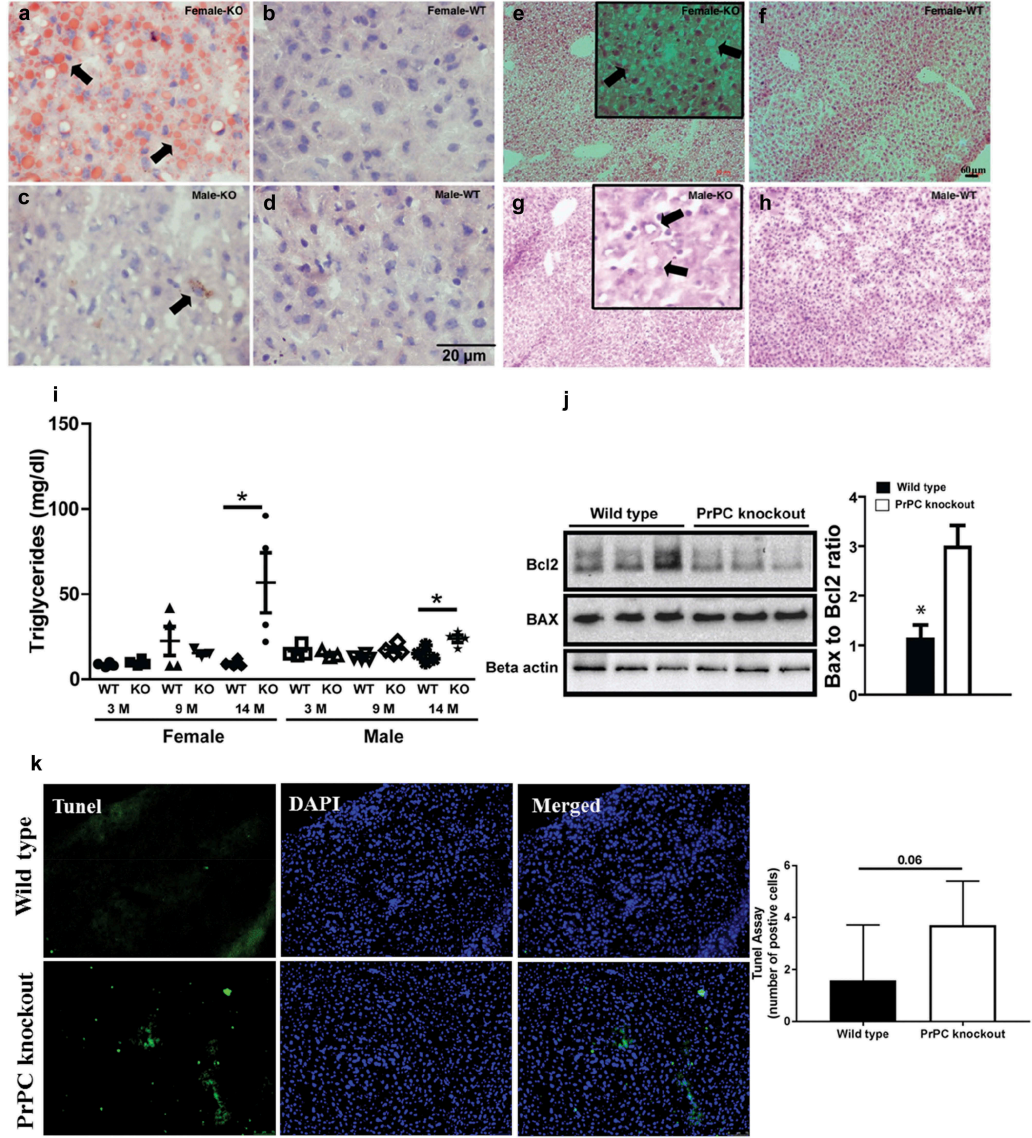
Lipid levels in PrPC knockout mice: Liver tissue stained with Sudan III (a-d), H and E stain (e-h) shows a higher fat deposition in the liver of 14 months female PrPC knockout mice (a and e) as compared to the wild type mice (b and f). Biochemical analysis shows a higher triglyceride concentration in 14 months PrPC knockout mice as compared to the wild type controls (i). Comparatively, the fat content in the liver of male PrPC knockout mice (c and g) was lower than the female group. Nuclei were stained with hematoxylin. Western Blot analysis showed a significant down-regulation of Bcl2 expression in the liver of PrPC knockout mice as compared to the wild type mice (j), while the expression of Bax was up-regulated in PrPC knockout liver with a net increase of Bax to Bcl2 ratio. (k) TUNEL assay in the 14-month-old PrPC knockout mice liver and WT controls. (3-month-old – 3 M, 9-month-old – 9 M, 14-month-old – 14 M).

Triglyceride content was measured in the liver of PrPC knockout mice as compared to the wild type. We found a significant up-regulation of triglyceride content (mg/dl) in the liver of 14 month-old mice, which indicated the presence of lipid metabolic syndrome or non-alcoholic fatty liver disease (NAFLD) in the ageing PrPC knockout mice (Figure 1(i)). In 14 month-old female PrPC knockout mice liver, the concentrations of triglycerides were higher than a male group of the same age.

### 2.4. *Effect of PrPC on in-vivo and* in vitro *fat content*

Further, we investigated the fat content by Sudan III and H and E staining in the liver of 14 month-old mice. We detected the fat globules in PrPC knockout mice liver, which confirmed the excessive fat deposition in the female PrPC knockout mice (Figure 1(a,e)) as compared to the wild type (Figure 1(b,f)). However, the fat globules were less in the liver of male PrPC knockout mice vs female group (Figure 1(c,g)) as compared to the wild type (Figure 1(d,h)).

**Figure 2.**
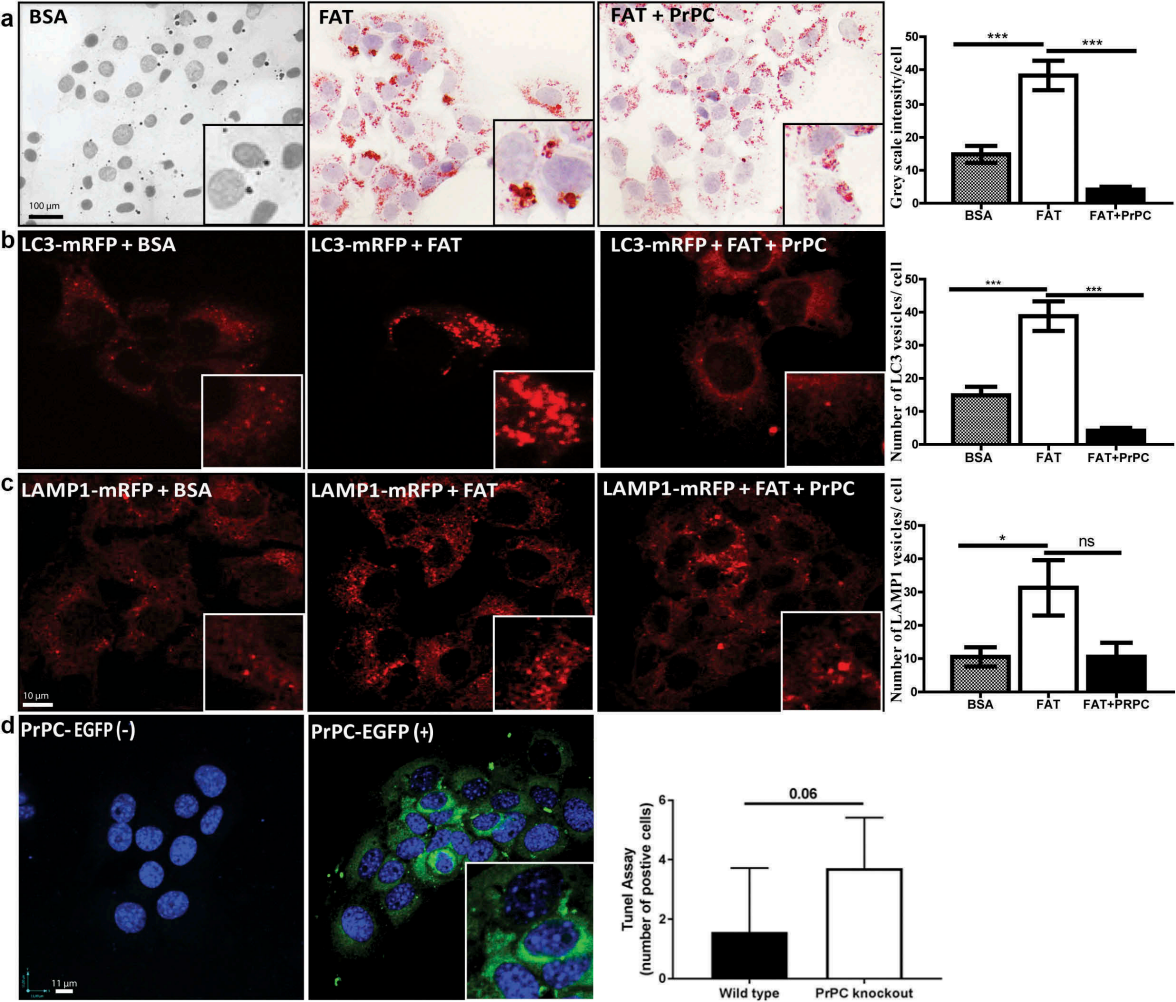
FAT accumulation and trafficking after PrPC- overexpression in AML12 cells: Oil-red-o staining in palmitate (FAT) treated AML12 cells shows that FAT accumulation is significantly reduced after PrPC over-expression in cells (a). The number of mRFP-LC3 vesicles are higher in FAT-treated cells as compared to BSA control and co-expression of PrPC-EGFP reduced the number of mRFP-LC3 vesicles (b). LAMP1-mRFP expression shows an increase in the number of vesicles in FAT-treated cells and there was a non-significant decrease in a number of LAMP1-mRFP vesicles after PrPC co-expression (c). A representative image shows an over-expression of PrPC-EGFP in AML12 cells.

Moreover, when AML12 cells were challenged with Saturated Free fatty acid (Palmitate) and it results in the accumulation of high-fat content in control cells in absence of PrPC over-expression. Cells transfected with plasmid DNA construct for EGFP-PrPC however, showed a significant reduction in fat accumulation as evidenced by the Oil-red-o staining of these cells (Figure 2(a)).

The close association of autophagy with metabolism in the context of steatosis has been shown in many studies [13]. There are clear evidences that free fatty acid accumulation in hepatocytes induces autophagy which plays an important role in fat metabolism [14]. This phenomenon is also called as lipophagy. The increased levels of LC3 protein and the presence of autophagy vacuoles are important markers for autophagy in the fatty liver [14,15]. Therefore, a number of mRFP-LC3 vesicles in AML12 cells treated with palmitate were analysed. We found a significant upregulation of the number of mRFP-LC3 vesicles (Figure 2(b)). However, when we compared these results with AML12 cells co-transfected with PrPC, we found a significant decrease in the number and size of mRFP-LC3 vesicles after palmitate treatment (Figure 2(b)). We also observed a significant increase in the number of mRFP-Lamp1 vesicles after FAT treatment and the number of same veiscles decreased after co-expression of PrPC, however; in this case, the differences were not significant (Figure 2(c)). Together, these results suggest the role of PrPC in the trafficking of fat and mobilization or degradation of excessive fat in these cells.

### 2.5. Quantitative gel-free proteomics of 14 months age mice

**Figure 3.**
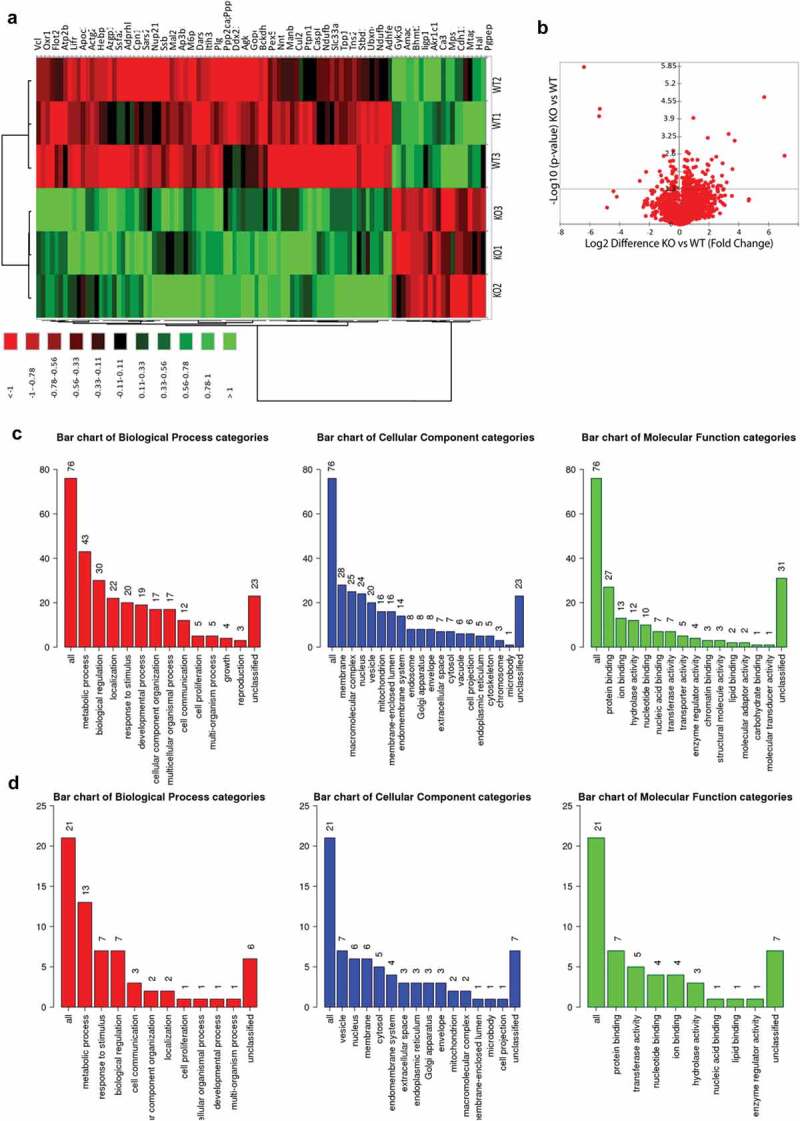
Proteome analysis of 14 months old PrPC knockout mice. a) Heat map showing the differentially regulated proteins in the liver of 14 month-old PrPC knockout mice (KO1, KO2, KO3) and WT (WT1, WT2, WT3) controls b) Volcano plot representing the all set of proteins detected in both mice groups and 102 proteins were found be significantly regulated with minimum – log10 p – value of 1.3 (0.05) Gene ontology classifications for biological process, cellular component and molecular functions of 76 up-regulated (c) and 21 down-regulated (d) proteins are shown graphically.

As we have observed significant changes in the amount of triglycerides specifically in 14 months age group. Therefore, we wanted to obtain a robust information of the proteome change in response to change in the excessive deposition of lipids. We analysed 3 samples per group (PrPC knockout and wild type) and 2718 proteins were detected by mass spec (Heat map Figure 3(a)). Eighty proteins were significantly up-regulated in PrPC knockout as compared to the wild type group (Table 2, Figure 3(b) – Volcano plot) and 21 proteins were significantly down-regulated with a minimum fold change of 1.5 or log2 difference of 0.58 (Table 3, Figure 3(b) – Volcano plot). The enrichment analysis by WEB-based GEne SeT AnaLysis Toolkit showed networks linked with non-alcoholic fatty liver disease (NAFLD), Hepatitis C and Lysosomal function (Table 4). Earlier proteomics datasets from 2DE analysis and chemical stainings showed a presence of higher lipid content in PrPC knockout liver. Therefore, proteome analysis by gel-free proteomics also showed a possible manifestation of non-alcoholic fatty liver disease, further indicating the accumulation of fat in the liver. Protein cluster which was found to be linked with NAFLD includes a set of four proteins: MAPK9, subunits of NADH dehydrogenase (ubiquinone) 1 beta subcomplex 5, 9 and 8. In addition, the previous study has reported a link of PrPC expression to Hepatitis C [16]. Regulated proteins associated with Hepatitis C observed in datasets include eukaryotic translation initiation factor 3, subunit E, protein phosphatase 2 (formerly 2A), catalytic subunit, beta isoform, and mitogen-activated protein kinase 9.

**Table 2.** Detailed list of statistically significant (P-value ≤ 0.05) protein dataset by gel-free proteomics of old-aged mice group (up-regulation): List included proteins up-regulated in the liver of 14-month-old of PrPC knockout mice as compared to the wild type. – log10 p-value is significant when higher than 1.3 (equivalent to P-value of 0.05) and minimum log2difference (fold change of 1.5) of + 0.58.

Sr. No.	T: Gene names	Mol. weight [kDa]	-Log10 p-value	log2Difference (Fold Change)
1	Ppp2ca; Ppp2cb	35.61	1.31	0.59
2	Plg	90.81	2.63	0.61
3	Atp2b1	134.75	1.41	0.61
4	Ap3b1	122.74	1.99	0.63
5	Mapk9	48.19	1.65	0.64
6	Pacsin3	48.58	1.36	0.66
7	Ndufb5	14.04	1.69	0.66
8	Vcl	116.72	1.52	0.67
9	Mal2	19.09	1.77	0.67
10	Nono	54.54	1.9	0.68
11	Gpt2	57.94	1.31	0.68
12	Agk	46.98	1.5	0.69
13	Csnk1a1	18.32	1.68	0.71
14	Ncbp1	91.93	1.31	0.75
15	Adhfe1	49.04	1.39	0.76
16	Calr4	49.45	1.55	0.8
17	Lpp	65.89	1.82	0.82
18	Bckdha	50.77	2.4	0.82
19	M6pr	31.17	1.62	0.85
20	Apoc3	10.92	1.34	0.86
21	Dars	57.15	3.95	0.9
22	Gstm2	25.72	2.11	0.92
23	Actg2	41.88	1.59	0.92
24	Ssb	47.76	1.66	0.94
25	Pxmp2	9.44	1.32	0.95
26	Adprhl2	39.41	1.58	0.98
27	Apol9a; Apol9b	33.3	1.31	0.99
28	Zzef1	328.27	1.41	0.99
29	Mar-01	37.98	1.31	1.03
30	Ssfa2	133.46	1.37	1.05
31	Sfpq	75.44	1.42	1.07
32	Asah1	44.67	2.14	1.08
33	Paf1	60.52	1.52	1.12
34	Ca5a	34.07	1.52	1.13
35	Sars2	58.32	1.61	1.15
36	Cpn1	51.85	2.4	1.18
37	Chd4	216.37	1.55	1.26
38	Ddx23	95.49	1.39	1.27
39	Sigirr	46.16	1.3	1.28
40	Cul2	86.88	1.63	1.3
41	Nup214	212.98	1.43	1.33
42	Tns2	152.01	1.64	1.44
43	Rabggta	64.99	1.3	1.47
44	Ptpn11	68.46	1.33	1.52
45	Hebp1	21.05	1.95	1.53
46	Flot2	47.12	1.45	1.54
47	Glyr1	60.44	1.39	1.55
48	Gopc	44.67	1.44	1.61
49	Pdcd6	21.87	1.36	1.65
50	Ndufb9	21.98	1.36	1.69
51	Dhtkd1	102.79	1.34	1.7
52	Azgp1	35.33	1.87	1.7
53	Snx17	7.58	1.56	1.71
54	Ascc2	85.65	1.87	1.73
55	Abcb8	78	1.3	1.81
56	Oxr1	91.74	2.23	1.85
57	Igha	36.72	2.01	1.86
58	Eif2b5	80.09	3.21	1.87
59	Ece1	87.07	1.49	1.89
60	Pex5	69.84	2.19	1.93
61	Vps51	86.19	1.37	1.94
62	Farp2	121.28	1.57	2.09
63	Ifitm3	14.95	2.41	2.14
64	Ndufb8	21.88	1.77	2.2
65	Slc33a1	61.08	1.43	2.23
66	Manba	100.85	1.62	2.25
67	Eif3e	52.22	2.51	2.45
68	Stbd1	36.13	1.38	2.5
69	Ubxn4	56.46	1.31	2.54
70	Casp6	31.6	1.45	2.58
71	Cela1	28.9	1.43	2.77
72	Tpp1	61.34	1.81	2.83
73	Golim4	79.9	2.27	2.87
74	Cav1	20.54	1.32	2.94
75	Tmem109	8.03	1.57	3.14
76	Itih3	99.36	3.35	3.27
77	Lifr	122.57	2.05	3.53
78	Ighg;Igh-1a	36.39	3.1	3.68
79	P01878	36.88	4.72	5.67
80	Nnt	113.84	2.55	7.03

**Table 3.** Detailed list of statistically significant (*P*-value ≤ 0.05) protein dataset by gel-free proteomics of old-aged mice group (down-regulation): List included proteins down-regulated in the liver of 14-month-old of PrPC knockout mice as compared to the wild type. – log10 p-value is significant when higher than 1.3 (equivalent to *P*-value of 0.05) and minimum log2difference (fold change of 1.5) of – 0.58.

Sr. No.	T: Gene names	Mol. weight [kDa]	-Log10 *p*-value	log2Difference (Fold Change)
1	Bhmt2	39.87	5.83	−6.44
2	A0A0R4J0I1	46.67	4.01	−5.42
3	Serpina1d	46	4.28	−5.37
4	Akr1 c19	37.05	1.62	−2.69
5	Tmed7	21.28	1.95	−2.27
6	C8b	66.23	1.41	−1.53
7	Pcyt1a	29.76	1.97	−1.35
8	Mpst	33.1	1.82	−1.26
9	Hal	72.26	1.58	−1.23
10	Luc7l3	51.45	1.3	−1.2
11	Yme1l1	80.03	1.63	−1.18
12	Ca3	29.37	1.59	−1.18
13	Cdh13	78.19	2.54	−1.08
14	Snrpf	9.73	1.71	−0.91
15	Amacr	41.7	1.61	−0.78
16	Pgpep1	12.12	1.33	−0.72
17	Gyk;Gk	59.83	2.34	−0.66
18	Mtap	31.06	1.3	−0.66
19	Iigp1	47.57	2.35	−0.61
20	Khk	32.74	2.16	−0.6
21	Arf5	20.53	1.35	−0.58

**Table 4.** Enrichment analysis of the proteins which are significantly up-regulated in PrPC knockout mice as compared to the wild type. ‘C’ stands for the number of reference genes in the category and ‘O’ stands for the number of genes in the user gene list and also in the category (file:///C:/Users/Arora/Desktop/Manuscript-submission/Figures/Proteomics/Up-regulated/Report_wg_result1517501423.html).

Description	C	O	Enrichment	*P*-value
Lysosome – Mus musculus (mouse)	124	5	0.65	0.001
Non-alcoholic fatty liver disease (NAFLD) – Mus musculus (mouse)	153	4	0.81	0.008
RNA transport – Mus musculus (mouse)	167	4	0.88	0.011
Aminoacyl-tRNA biosynthesis – Mus musculus (mouse)	44	2	0.23	0.022
Sphingolipid signalling pathway – Mus musculus (mouse)	124	3	0.65	0.027
Oxidative phosphorylation – Mus musculus (mouse)	134	3	0.72	0.033
Hepatitis C – Mus musculus (mouse)	136	3	0.72	0.034
Parkinson’s disease – Mus musculus (mouse)	144	3	0.76	0.040
Renal cell carcinoma – Mus musculus (mouse)	67	2	0.35	0.048

### 2.6. Regulation of PPARα, ACC and FAS genes in the liver of PrPC knockout mice by qPCR

**Figure 4.**
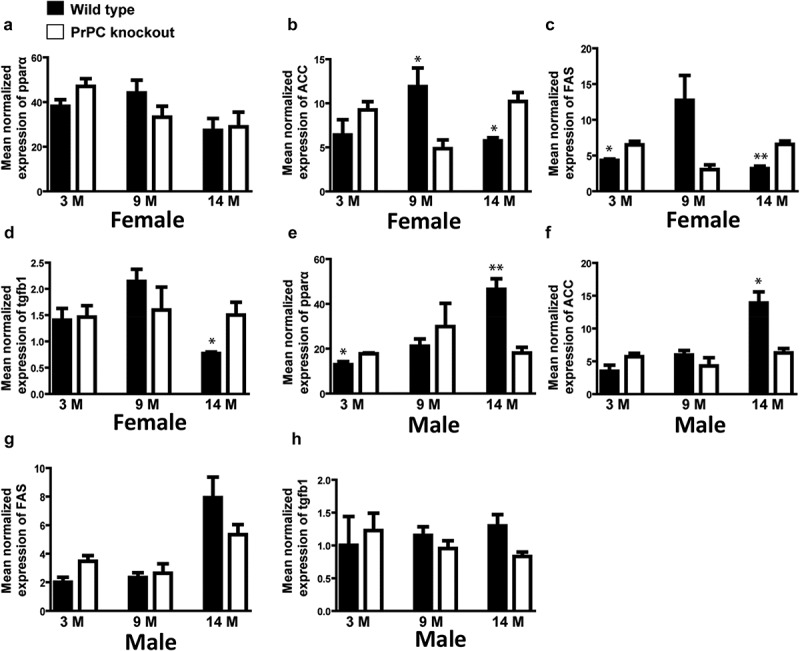
Gender-dependent regulation of hepatic mRNA expression of PPARα, ACC, FAS and tgfb1 in PrPC knockout mice: The mRNA expression of ACC and FAS genes was significantly up-regulated in the 14 month-old female PrPC knockout mice (b and c), while the expression of ACC in the 14 month-old male PrPC knockout mice was significantly down-regulated (f) and no significant differences in FAS mRNA expression was observed in the male group (g). The expression of PPARα mRNA was significantly down-regulated in 14-month-old male PrPC knockout mice (e) while there was no regulation in the female group (a). The mRNA expression of tgfb1 was significantly up-regulated only in the female PrPC knockout mice (d). (3-month-old – 3 M, 9-month-old – 9 M, 14-month-old – 14 M).

**Figure 5.**
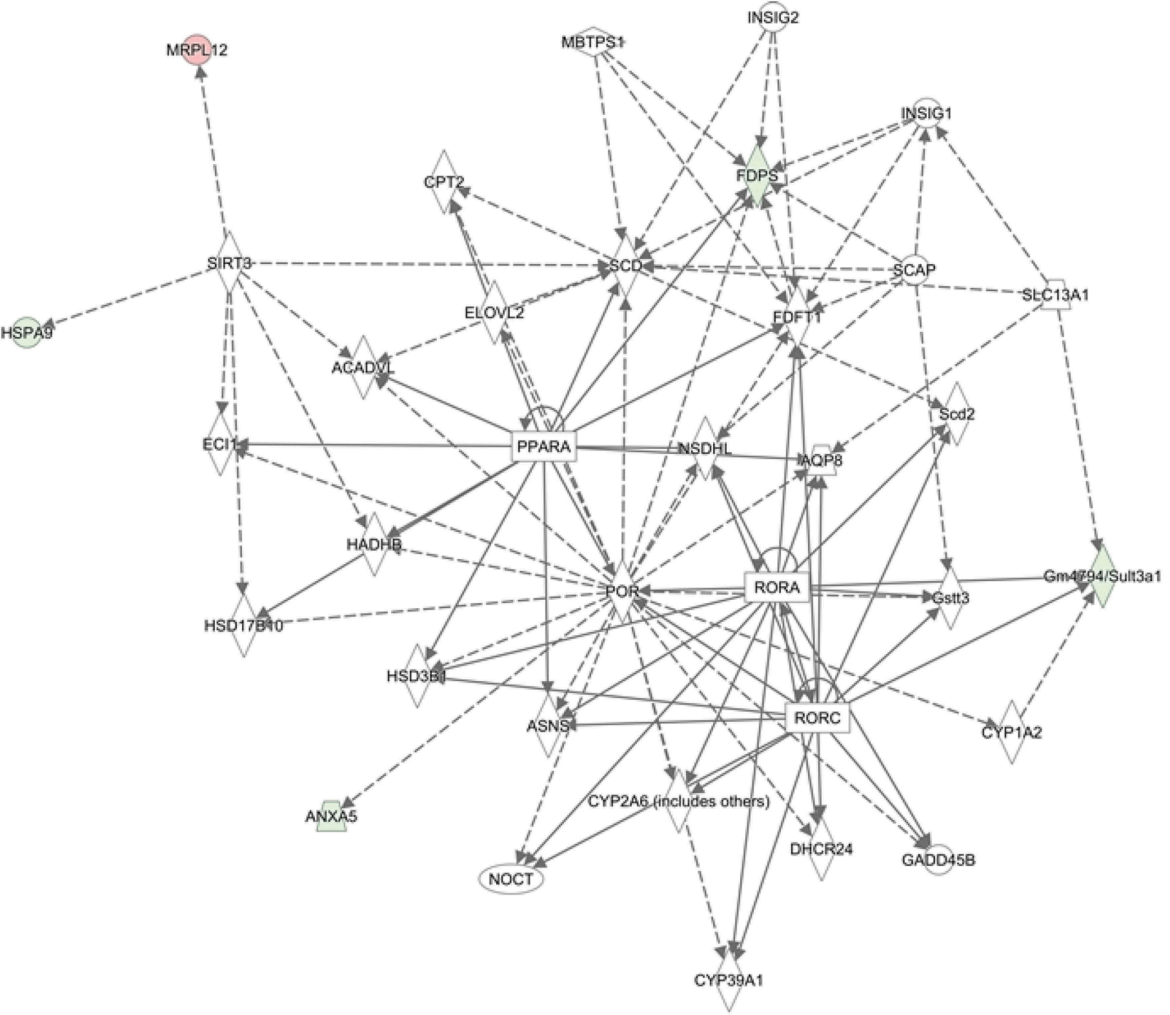
IPA software network 1 – Functional network analysed by comparing the proteome dataset of PrPC knockout mice liver and wild type (both genders and all age groups) (Details – Table 2S). Coloured proteins labels were found to be regulated in our proteome dataset and uncoloured labels are predicted to be linked by the ingenuity software. The network is associated with Lipid metabolism, Small molecular biochemistry, Vitamin and Mineral metabolism. PPARA (PPARα) gene has high connectivity in the network with clusters of genes which are reported to be involved in lipid metabolism. For example, Amine sulfotransferase (Gm4794/Sult3a1) is found to be 3.01-fold down-regulated in 14 months female PrPC knockout mice as compared to the wild type and it has already reported being down-regulated in liver steatosis [39].

Further, IPA software analysis predicted a detailed list of possible regulated genes linked with lipid metabolism (Table 2S, supporting information). Among them, the crucial transcriptional factor PPARα is a regulator of fatty acid β-oxidation (Figure 5). mRNA expression of PPARα in the 14 month-old PrPC knockout mice liver was analysed by qPCR and we observed a down-regulation of PPARα in the male PrPC knockout mice (Figure 4(e)), while no regulation of PPARα was observed in the female PrPC knockout mice (Figure 4(a)). We further analysed the expression of the enzymes for *de novo* fatty acid synthesis, acetyl CoA carboxylase gene (ACC) and fatty acid synthase (FAS) by qPCR. We found a significant up-regulation of hepatic ACC and FAS genes expression in 14 month-old female PrPC knockout mice (Figure 4(b,c)) while the expression of ACC in male PrPC knockout mice was down-regulated (figure 4(f)) and no significant differences of FAS mRNA expression was observed in the male group (Figure 4(g)).

### 2.7. Evidences of metabolic disease in the liver of female PrPC knockout mice

To further validate, if any pathological manifestation is due to higher accumulation of fat, we analysed the expression of tgfb1 by qPCR, which is a marker of fibrogenesis. We found a significant up-regulation of tgfb1 in the 14 month-old female PrPC knockout mice liver (Figure 4(d)) but not in the male group (Figure 4(h)).

Furthermore, the imbalance of pro-apoptotic protein Bax and anti-apoptotic protein Bcl2 has been reported to contribute to hepatocyte apoptosis during the pathogenesis of excessive fat accumulation [17]. Hence, we further analysed the expression of Bax and Bcl2 in the liver of PrPC knockout female mice by Western Blot because excessive fat accumulation was observed only in the female group. We observed a significant increase of Bax to Bcl2 ratio in female PrPC knockout mice liver as compared to the wild type (Figure 1(j)). Further quantification of apoptotic cell death by TUNEL staining showed near significant (P = 0.06) increase in the number of apoptotic cells in PrPC knockout cells as compared to wild type.

## 3. Discussion

Our current study was focused on the illustration of PrPC function in the liver. The proteome analysis of PrPC knockout mice liver pointed to the regulation of lipid metabolism in the 14 month-old PrPC knockout mice. Further *in vitro* experiments validated that PrPC regulates the fat accumulation in hepatic AMP12 cell line with parallel regulation of autophagy.

Excessive fat deposition observed in the liver of ageing PrPC knockout mice could be because of non-alcoholic fatty liver disease (NAFLD). However, to confirm if excessive fat accumulation is due to NAFLD needs further in detailed pathological as well as serological investigations. In general, the incidences of fatty liver disease are increasing among the ageing population, which was earlier demonstrated, using senescence-accelerated prone mice (SAMP8) [18]. The deposition of excessive fat in the liver of PrPC knockout mice showed that lipid metabolism is affected in absence of PrPC; maybe because of multiple possibilities including the enhanced release of non-esterified fatty acids from adipose tissue (lipolysis), increased *de novo* fatty acid synthesis (lipogenesis), decreased beta-oxidation of fatty acids or due to trafficking defect [19].

### 3.1. Regulation of genes involved in lipid metabolism

Ingenuity pathway analysis of 2DE proteome results predicted the involvement of PPARα as a possible regulatory gene linked with lipid metabolism. PPARα is a transcription factor which regulates the beta-oxidation of fatty acids. The down-regulation of PPARα causes fat accumulation due to decreased oxidation of fatty acids [20,21]. Surprisingly, the down-regulation of PPARα gene expression was observed only in the male PrPC knockout mice, while the excessive fat deposition was observed in the female group. This may be due to PrPC dependent differential regulatory mechanisms of lipid metabolism by PPARα in the female and male liver [22]. Therefore, it prompted us to look for other genes associated with lipid metabolism.

Acetyl CoA-carboxylase (ACC) is a rate-limiting enzyme in *de novo*-lipogenesis while fatty acid synthase (FAS) is a key enzyme involved in terminal catalytic steps of fatty acid synthesis [23]. The expression of ACC and FAS are known to be increased in NAFLD [24,25]. We found a significant up-regulation of ACC as well as FAS mRNA expression in the 14 months female PrPC knockout mice. Therefore, higher expression of ACC and FAS mRNA indicates an increased *de novo* fatty acid synthesis in the liver of PrPC knockout mice. The above observation explains a metabolic regulatory mechanism, which leads to excessive fat deposition in the liver in the absence of PrPC.

This age-dependent shift in the genes expression of lipid metabolism in PrPC knockout mice co-relates with the findings showed up- and down-regulation of FAS and SREBP-1 c mRNA expressions, respectively, in senescence-accelerated prone mice (SAMP8) manifested with NAFLD [18]. Therefore, excessive fat accumulation in the ageing PrPC knockout mice shows that PrPC may have an anti-ageing effect on the age-dependent shift of gene expression involved in lipid metabolism.

Another observation from the current study showed that PrPC has a differential effect on lipid metabolism in the female and male groups, possibly because of gender-dependent expression of PrPC in the liver of wild type mice. The wild type female mice have a higher expression of PrPC in the liver as compared to the male [12]. Apparently, PrPC has a more significant role in the liver of female mice, because as we have observed, the knockout of PrPC gene affected female mice severely than male mice. The understanding of the hormonal influence on the gender-dependent expression of PrPC on lipid metabolism is beyond the scope of the current study.

### 3.2. Evidences of metabolic disease linked with excessive fat accumulation

Gel-free proteome analysis of 14 month-old PrPC knockout mice found an up-regulation of MAPK9 or JNK2, NADH dehydrogenase (ubiquinone) 1 beta subcomplex subunits 5, 8 and 9. All four genes are part of NAFLD network of KEGG PATHWAY. It is reported that JNK2 promotes lipoapoptosis in hepatic steatosis and acts as a pro-survival factor in lipid toxicity [14]. Therefore, the up-regulation of JNK2 indicates increased lipotoxicity due to increased fat accumulation in the liver of PrPC knockout mice. Further, up-regulation of a cluster of three NADH dehydrogenase (ubiquinone) 1 beta subcomplex subunits was observed in the liver of PrPC knockout mice and all three subunits are part of complex 1 of the mitochondrial respiratory chain [26]. Fatty acids undergo mitochondrial β-oxidation to release acetyl-CoAs which generates NADH and NADH undergo oxidative phosphorylation through mitochondrial respiratory chain [26]. Upregulation of NADH dehydrogenase subunits in the liver of PrPC knockout mice indicates a compensatory response because of increased load of NADH which may be additionally produced due to breakdown of excess fat as observed in the liver in absence of PrPC. Interestingly, we also observed a significant up-regulation of APOC3 protein in PrPC knockout mice which regulates triglyceride metabolism in multiple ways, including hepatic- and lipoprotein lipase-mediated TG hydrolysis, by promoting hepatic VLDL-TG production and by inhibiting uptake and clearance of TG-rich lipoprotein remnants [27]. It is already known that animal models with the over-expression of APOC3 lead to excessive triglycerides levels [27]. Therefore, the up-regulation of APOC3 in the liver of 14 months age PrPC knockout mice as compared to controls may be one of the contributing metabolic factors that leads to excessive fat deposition in absence of PrPC [27].

As mentioned, excessive fat accumulation may be the case of NAFLD which could further be associated with cell death, fibrosis, or liver cirrhosis [28]. Although, the expression of Bax was not significantly increased; however, a net increase of Bax/Bcl2 ratio was found due to a significant decrease of Bcl2 in the liver of 14 month-old female PrPC knockout mice as compared to the wild type by Western Blot. Bax and Bcl-2 are anti- and pro-apoptotic members, respectively, of the bcl-2 family [29] and higher Bax to Bcl-2 ratio is increased in the progression of non-alcoholic fatty liver disease with the manifestation of apoptosis [30]. However, it is possible that cell death in PrPC knockout old age mice is mild as further evidenced by TUNEL cell death assay which showed a non-significant, though near to significant increase in the number of apoptotic cells with a p-value of 0.06.

Tgfb1 is a growth factor with anti–inflammatory and pro-fibrogenic properties [31]. Also, tgfb1 is known as an early phase marker of NAFLD progression into non-alcoholic steatohepatitis NASH [32,33]. We showed an up-regulation of tgfb1 mRNA level in the 14 month-old female PrPC knockout mice liver. Hence, it evidenced that NAFLD in female PrPC knockout mice liver may have a fibrogenic response.

### 3.3. Regulation of FAT accumulation in AML12 cells

To understand the mechanism of fat accumulation in the liver of PrPC knockout mice; we analysed the fat levels in AML12 liver cell line in the presence of PrPC overexpression. Interestingly, fat analysis in over-expressing PrPC AML12 cells showed significantly decreased FAT content; validating the role of PrPC in regulating the lipid metabolism. However, as the liver of PrPC knockout mouse showed an increased fat accumulation that could be due to a lack of clearance of accumulated fat in hepatocytes in the absence of PrPC. Further, the treatment of fatty acids significantly decreased the number and size of accumulated mRFP-LC3 marked autophagy vesicles, an effect which is already known [34]. Interestingly, PrPC overexpression reversed the accumulation of mRFP-LC3 marked autophagy vesicles. Previous studies already reported that deficiency of PrP (C) may impair autophagic flux in oxidative stress [16] and overexpression of PrPC inhibits autophagy-mediated lipid accumulation in 3T3-L1 adipocytes [4] which correlates with our findings that overexpression of PrPC not only reversed the accumulated autophagosomes induced by fat treatment but also reduced the excessive fat in the hepatic cells. Therefore, our observations showed that PrPC is involved in the regulation of intracellular fat mobilization.

### 3.4. Concluding Remarks

In the current study, with the help of proteomics and cell biology approach, the significance of PrPC in lipid metabolism in ageing liver is shown. The excessive hepatic fat deposition is mainly observed in the female PrPC knockout mice, with the evidences of non-alcoholic fatty liver disease. Cell culture experiments in the hepatic AML12 cell line further showed that PrPC regulates the intracellular fat levels.

## 4. Material and methods

### 4.1. Animals

PrP knockout mice from 3, 9 and 14 months-old from both sexes (4 mice per group) were derived from Zurich I with a mixed genetic background (129/Sv and C57BL/6) and were generated as previously described by Bueler et al. [35]. As control mice, we used WT mice from the same genetic background. All protocols used were in accordance with the ethical rules for animal experimentation.

### 4.2 Two-dimensional gel electrophoresis (2DE)

After the mice were sacrificed, liver tissues were dissected, snap-frozen into liquid nitrogen and stored at −80°C until further use. The tissues were lysed in lysis buffer (7 M urea, 2 M thiourea, 4% CHAPS, 20 µl/ml ampholytes, 10 mg/ml DTT, protease, and phosphatase inhibitors) for 5 min at 50-hertz frequency and incubated overnight at 4°C. Lysed tissue samples were centrifuged at 14,000 rpm for 20 min at 4°C and supernatants were obtained for further experiments.

Protein concentrations were measured by the Bradford protein estimation method (Bio-Rad standard protocol) and lysates containing 120 µg of protein were diluted into 325 µL rehydration buffer (7 M urea, 2 M thiourea, 4% CHAPS, 0.2% 3–10 bio-Lytes and 65 mM DTT) and loaded on a ReadyStrip (IPG nonlinear pH 3–10, 17 cm strip – Bio-Rad). After 12 h of active rehydration at 50 volts (V), isoelectric focusing was started at 500 V for 1 h, followed by ramping at 1000 V for 1 h and 5000 V for 2 h. The final focusing was conducted at 8000 V, reaching a total of 60,000 V hours (PROTEAN IEF CELL, Bio-Rad). Then, the strips were equilibrated 2 times for 20 min in buffer containing 6 M urea, 2% SDS, 30% glycerine, and 0.375 M Trisph, pH 8.8, supplemented with 2% DTT in the first and with 2.5% Iodoacetamide (IAA) in the second equilibration step. SDS-PAGE was performed overnight at 4°C with homogeneous 12% polyacrylamide gel using a PROTEAN II XL Vertical Electrophoresis Cell (Bio-Rad).

2D gels were stained with silver stain and gel images were scanned at 300 dpi with canon LiDe 110 scanner. Protein spot abundances from 48 liver proteome gel images (3, 9, 14 month-old, wild type and PrPC knockout from both sexes) were analysed using the Delta2D software (v. 3.6) (DECODON). The differences in spot abundance analysed by Delta2D software were statistically evaluated using unpaired Student’s t-test. Mean and standard deviation were calculated from four sets of experiments. A protein spot was considered as differentially regulated when its densitometric analyses showed at least 1.5-fold change in abundance and when the p-value was <0.05 in unpaired Student’s t-test.

### 4.3. Mass spectrometry

The protein spots from three gel replicate with pooled samples from all groups were used for mass spectrometry identification analysis. Gel plugs containing proteins were manually excised from silver-stained gels and subjected to in-gel digestion. Ramljak et al. [36] give the detailed protocol of this procedure. In-gel digested peptides were chromatographically separated (Reversed-Phase-C18 nanoflow chromatography, using a 15-min linear gradient on an Easy nLC-1000 nanoflow chromatography system (Thermo Fisher Scientific, Dreieich, Germany)) and analysed by Q Exactive hybrid quadrupole/orbitrap mass spectrometry system operated under Excalibur v2.4 software (Thermo Fisher Scientific). For database searching, tandem mass spectra were extracted using Raw2MSM v1.17 software (Max Planck Institute for Biochemistry, Martinsried, Germany). All MS/MS samples were analysed using Mascot (Matrix Science, London, UK; version 2.4.1) set up to search the UniProt/SwissProt database (release 02/14 filtered for *Mus musculus*, 16,665 entries) with mass tolerances of 5 ppm for precursors and 0.020 Da for fragments, respectively. The searching criteria were set with one missed cleavage by trypsin allowed and protein modifications set to methionine oxidation and carbamidomethylcysteine when appropriate. The proteome datasets obtained by mass spectrometry identification was further analysed by Inguniety pathway analysis (Details are provided in supporting information).

### 4.4. Gel-free proteomics

To further validate the results obtained from 2DE, we further selected samples of old age of PrPC knockout mice and wild type to perform gel-free proteomics analysis. We prepared the samples similar to as prepared for 2DE and sent for proteomics analysis at Proteomics Facility, CECAD, Köln. Proteins were considered regulated with a p-value less than 0.5 with a minimum fold change of 1.5

### 4.5. Analysis of fat content

Liver tissues from the mice were lysed in PBS and supernatants were obtained after centrifugation at 14,000 rpm for 20 min. Triglycerides concentrations from the tissue lysates were measured in milligram per decilitre (Centre facility, Department of clinical chemistry, UMG, Göttingen). The kit was used for determining the triglyceride concentration (REF: 7D74-21, http://www.ilexmedical.com/files/PDF/Triglyceride_ARC_CHEM.pdf.). The liver tissue sections (5 µm thickness, by Leica cryostat 3050) glass slides were further stained with Sudan III and H and E stains, followed by microscopy with the bright field.

### 4.6. Western blotting

Liver samples were homogenized and lysed with the same protocol and procedure as for 2D gel electrophoresis. An amount of 75 µg of the liver samples were separated on 12% SDS-PAGE gels and transferred to PVDF membranes. The membranes were blocked with 5% skimmed milk in phosphate buffer saline with 0.05% Tween 20 (PBST) for 1 h at room temperature. Subsequently, membranes were incubated overnight at 4°C with the following primary antibodies: rabbit anti-Bcl2 (1:1000, Cell signalling), mouse anti-Bax (1:1000, Cell Signalling), mouse anti-Beta-actin (1:2000, Abcam). Thereafter, membranes were washed with PBST and incubated for 1 h at room temperature with corresponding horseradish peroxidase-conjugated secondary antibodies: goat anti-mouse (1:7500 Abcam), goat anti-rabbit (1:10,000, Jackson Immunoresearch). The immunoreactivity was detected after immersing the membranes in enhanced chemiluminescence (ECL) solution and the signal was detected using Chemi DOC XRS+ (BIO-RAD).

### 4.7. Quantitative real-time PCR (qPCR)

Sample preparation and total RNA extraction from liver tissue were carried out as per instructions from a commercial kit (mirVana isolation kit Ambio, Austin, TX). The retrotranscriptase reaction of the RNA samples was carried out with the High Capacity cDNA Archive kit (Applied Biosystems, US) following the protocol provided by the manufacturer and using the Gene Amp® 9700 PCR System thermocycler (Applied Biosystems, USA). Roche LightCycler 480 detector instrument was used for PCR amplification and detection. Parallel amplification reactions for each sample were performed using the 20 × TaqMan Gene Expression Assays (Applied Biosystems) and 2 × TaqMan Universal PCR Master Mix (Applied Biosystems). Different steps were as follows: denaturation-activation cycle (50°C for 2 min, 95°C for 10 min) followed by 40 cycles of denaturation-annealing-extension (95°C, 15 s; 60°C for 1 min). mRNA levels were calculated using the LightCycler 480 software. Data analysis was done by ΔΔCt (cycle threshold) method to determine the gene expression values as fold changes between PrPC knockout and wild type groups, which were normalized by the relative expression of a housekeeping gene (HPRT). Commercial gene expression assays (Life technologies) comprise those for HRPT (Mm00446968_m1), PPARα (Mm00440939_m1), ACC (Mm01304257_m1), FAS (Mm00662319_m1) and tgfb1 (Mm01178820_m1).

### 4.8. Cell culture

Lamp1-RFP -, pmRFP-LC3, and EGPN1 plasmids were purchased from Addgene (provided by the lab of Dr. Ira Milosevic, ENI, Goettingen, Germany). PrPC gene was amplified from pCIneoPRNP vector [37] and cloned into EGFP-N1 for the current study. Lamp1 was cloned into pmRFP vector. AM12 cells were transfected with plasmid DNA expressing cloned genes (EGFP-N1 -PrPC-EGFP, pmRFP-LC3 and pmRFP-Lamp1). After 15-h post-transfection cells were treated with fatty acids (FAT) and BSA controls for 8 h. Treatments were prepared according to the method which is reported [38]. For visualization of FAT globules, cells were fixed and stained with oil red staining followed by light microscopy. For imaging of mRFP-LC3 and mRFP-Lamp1 markers, cells were fixed and observed directly under a confocal fluorescence microscope. 6 to 7 cells per image were used for quantitative analysis by image j.

### 4.9. Statistical analysis

All statistical analysis were performed using unpaired Sudent's t test *P < 0.05, **P < 0.01 and ***P < 0.001 and graphs were prepared with Prism-GraphPad software. Error bars in the graphs represents standard error of mean (SEM).

## Abbreviations

PrPC: Cellular prion protein
PrPSc: Prion protein Scrapie
TSE: Transmissible spongiform encephalopathies
2DE: Two-dimensional gel electrophoresis
IPA: Ingenuity pathway analysis
NAFLD: Non-alcoholic fatty liver disease
