# Supplementary material for: The role of cellular prion protein in lipid metabolism in the liver
Source: Prion. 2020 Mar 5;14(1):95–108. doi: 10.1080/19336896.2020.1729074 (PMC7153832; doi:10.1080/19336896.2020.1729074)
Supplement: Supplemental Material [file kprn-14-01-1729074-s002.docx]

**Supporting information**





**Fig.S1. 2DE gel map of liver PrPC knockout mice vs wild type mice liver (female):** Differentially regulated sports in 3, 9 and 14 month-old PrPC knockout mice **(A, C and E, respectively)** vs wild type mice liver **(B, D and F, respectively)**.


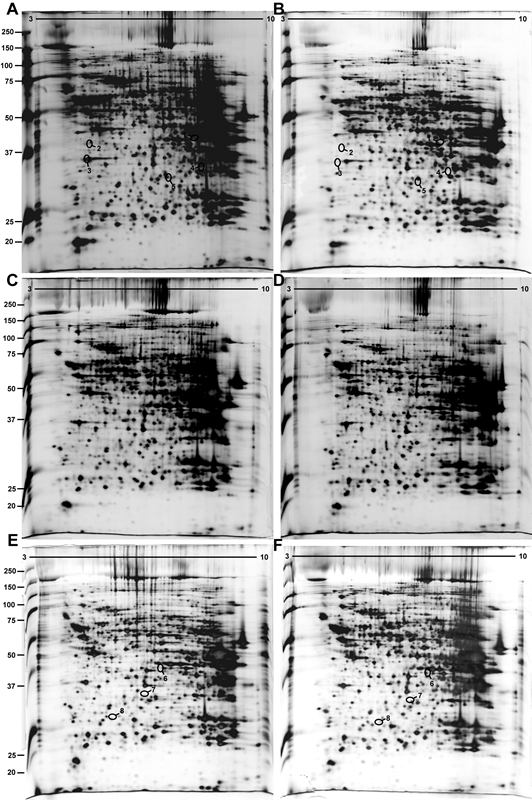


**Fig.S2. 2DE gel map of liver PrPC knockout mice vs wild type mice liver (male):** Differentially regulated sports in 3 and 14 month-old PrPC knockout mice **(A and C, respectively)** vs wild type mice liver **(E and F, respectively).** However, no significantly regulated spots were found in 9 month-old age male group **(C and D).**

**Table 1S – Detailed list of statistically significant (P value ≤ 0.05) dataset from mass spectrometry analysis (included multiple proteins detected by mass spectrometer in each spot):**

| **Sr.**  **no.** | **Protein name** | **Accession number** | **MW (kDa)** | **Coverage %** | **Score** | | **Fold Change** | ***P*-value** | ***pI*** |
| --- | --- | --- | --- | --- | --- | --- | --- | --- | --- |
|  | **Male - 3 month-old mice** |  |  |  | |  |  |  |  |
| 1 | Isoamyl acetate-hydrolyzing esterase 1 homolog | Q9DB29 | 27.975 | 36.90% | | 24 | 1.55 ↓ | 0.018 | 5.34 |
| 2 | Serine-threonine kinase receptor-associated protein | Q9Z1Z2 | 38.443 | 40.30% | | 26 | 1.53 ↓ | 0.014 | 4.99 |
| 3 | Regucalcin | Q64374 | 33.407 | 56.90% | | 155 | 1.59 ↓ | 0.031 | 5.16 |
|  | Thioredoxin-like protein 1 | Q8CDN6 | 32.237 | 45.70% | | 34 |  |  | 4.84 |
| 4 | Not identified |  |  |  | |  | 4.65 ↓ | 0.025 |  |
| 5 | Propionyl-CoA carboxylase alpha chain, mitochondrial | Q91ZA3 | 79.922 | 17.70% | | 17 | 1.58 ↓ | 0.04 | 6.04 |
|  | **Male - 14 month-old mice** |  |  |  | |  |  |  |  |
| 6 | U5 small nuclear ribonucleoprotein 200 kDa helicase | Q6P4T2 | 244.553 | 12.30% | | 36 | 1.77 ↓ | 0.012 | 5.73 |
|  | S-methylmethionine--homocysteine S-methyltransferase BHMT2 | Q91WS4 | 39.872 | 44.60% | | 36 |  |  | 6.10 |
|  | Pre-mRNA-processing-splicing factor 8 | Q99PV0 | 273.625 | 6.85% | | 24 |  |  | 8.95 |
|  | NADH dehydrogenase [ubiquinone] flavoprotein 2, mitochondrial | Q9D6J6 | 27.286 | 49.20% | | 24 |  |  | 5.31 |
|  | 116 kDa U5 small nuclear ribonucleoprotein component | O08810 | 109.363 | 16.10% | | 22 |  |  | 4.86 |
|  | Transaldolase | Q93092 | 37.389 | 24.30% | | 18 |  |  | 6.57 |
| 7 | 3-mercaptopyruvate sulfurtransferase | Q99J99 | 33.023 | 67.30% | | 68 | 6.70 ↑ | ≤0.001 | 6.12 |
|  | Delta(3,5)-Delta(2,4)-dienoyl-CoA isomerase, mitochondrial | O35459 | 36.119 | 45.00% | | 54 |  |  | 6.01 |
|  | 3-hydroxyanthranilate 3,4-dioxygenase | Q78JT3 | 32.804 | 54.90% | | 48 |  |  | 6.10 |
|  | L-xylulose reductase | Q91X52 | 25.745 | 34.00% | | 16 |  |  | 6.83 |
| 8 | Not identified |  |  |  | |  | 1.53 ↓ | 0.046 |  |
|  | **Female - 3 month-old mice** |  |  |  | |  |  |  |  |
| 9 | Farnesyl pyrophosphate synthase | Q920E5 | 40.583 | 42.80% | | 105 | 2.28 ↓ | 0.02 | 5.48 |
|  | Isocitrate dehydrogenase [NAD] subunit alpha, mitochondrial | Q9D6R2 | 39.639 | 34.20% | | 18 |  |  | 5.59 |
|  | Calponin-3 | Q9DAW9 | 36.430 | 33.60% | | 17 |  |  | 5.46 |
| 10 | Annexin A5 | P48036 | 35.754 | 59.90% | | 182 | 2.16 ↓ | 0.006 | 4.82 |
|  | Elongation factor 1-delta | P57776 | 31.293 | 21.70% | | 12 |  |  | 4.91 |
| 11 | Alpha-soluble NSF attachment protein | Q9DB05 | 33.191 | 70.5% | | 146 | 2.14 ↓ | 0.013 | 5.30 |
|  | 40S ribosomal protein S3a | P97351 | 29.885 | 40.2% | | 25 |  |  | 9.75 |
|  | Cathepsin Z | Q9WUU7 | 33.996 | 20.6% | | 22 |  |  | 5.31 |
|  | Annexin A4 | P97429 | 35.918 | 33.5% | | 18 |  |  | 5.42 |
|  | Apolipoprotein E | P08226 | 35.866 | 25.1% | | 9 |  |  | 5.46 |
| 12 | 3-mercaptopyruvate sulfurtransferase | Q99J99 | 33.023 | 67.30% | | 68 | 3.54 ↑ | 0.04 | 6.12 |
|  | Delta(3,5)-Delta(2,4)-dienoyl-CoA isomerase, mitochondrial | O35459 | 36.119 | 45.00% | | 54 |  |  | 6.01 |
|  | 3-hydroxyanthranilate 3,4-dioxygenase | Q78JT3 | 32.804 | 54.90% | | 48 |  |  | 6.10 |
|  | L-xylulose reductase | Q91X52 | 25.745 | 34.00% | | 16 |  |  | 6.83 |
| 13 | Vacuolar protein sorting-associated protein 29 | Q9QZ88 | 20.496 | 44.50% | | 39 | 2.14 ↑ | 0.006 | 6.29 |
|  | Probable imidazolonepropionase | Q9DBA8 | 46.489 | 24.20% | | 15 |  |  | 6.47 |
| 14 | 14-3-3 protein gamma | P61982 | 28.303 | 36.0% | | 21 | 2.66 ↑ | 0.017 | 4.80 |
| 15 | 39S ribosomal protein L12, mitochondrial | Q9DB15 | 21.709 | 55.70% | | 48 | 2.07 ↑ | 0.004 | 5.35 |
|  | Ferritin heavy chain | P09528 | 21.067 | 31.30% | | 16 |  |  | 5.53 |
|  | **Female - 9 month-old mice** |  |  |  | |  |  |  |  |
| 16 | Histidine ammonia-lyase | P35492 | 72.259 | 14.90% | | 30 | 2.05 ↓ | 0.039 | 5.94 |
|  | Aminoacylase-1 | Q99JW2 | 45.781 | 35.30% | | 26 |  |  | 5.89 |
| 17  18 | Leukocyte elastase inhibitor A  Sorbitol dehydrogenase  Alcohol dehydrogenase class-3  Apolipoprotein A-I  3-mercaptopyruvate sulfurtransferase | Q9D154  Q64442  P28474  Q00623  Q99J99 | 42.577  38.249  39.547  30.615  33.023 | 44.10%  27.50%  16.60%  16.30%  67.30% | | 59  14  12  6  68 | 2.19 ↓  2.53 ↑ | 0.009  0.019 | 5.85  6.60  7.11  5.31  6.12 |
|  | Delta(3,5)-Delta(2,4)-dienoyl-CoA isomerase, mitochondrial  3-hydroxyanthranilate 3,4-dioxygenase  L-xylulose reductase | O35459  Q78JT3  Q91X52 | 36.119  32.804  25.745 | 45.00%  54.90%  34.00% | | 54  48  16 |  |  | 6.01  6.10  6.83 |
|  |  |  |  |  |  |  |  |  |  |
|  |  |  |  |  |  |  |  |  |  |
| 19 | Proteasome subunit beta type-3 | Q9R1P1 | 22.96 | 55.60% | | 103 | 2.28 ↑ | 0.04 | 6.13 |
|  | Triosephosphate isomerase | P17751 | 32.191 | 49.80% | | 34 |  |  | 5.56 |
|  | Maleylacetoacetate isomerase | Q9WVL0 | 24.27 | 65.70% | | 28 |  |  | 7.68 |
|  | Ig kappa chain C region | P01837 | 11.778 | 56.60% | | 28 |  |  | 5.56 |
|  | Cyclin-dependent kinase inhibitor 1B/P2z | P46414 | 22.193 | 33.00% | | 6 |  |  | 6.54 |
|  | **Female - 14 month-old mice** |  |  |  | |  |  |  |  |
| 20  21 | Not identified  Stress-70 protein, mitochondrial | P38647 | 73.462 | 69.50% | | 507 | 1.82 ↑  1.56 ↓ | 0.012  0.029 | 5.44 |
|  | Lamin-B2 | P21619 | 67.319 | 49.30% | | 64 |  |  | 5.36 |
|  | Heat shock cognate 71 kDa protein | P63017 | 70.873 | 29.60% | | 25 |  |  | 5.37 |
| 22 | Actin, cytoplasmic 1 | P60710 | 41.738 | 37.60% | | 19 | 4.96 ↓ | 0.0004 | 5.29 |
| 23 | Leukocyte elastase inhibitor A | Q9D154 | 42.577 | 44.10% | | 59 | 2.23 ↑ | 0.012 | 5.82 |
|  | Sorbitol dehydrogenase | Q64442 | 38.249 | 27.50% | | 14 |  |  | 6.60 |
|  | Alcohol dehydrogenase class-3 | P28474 | 39.547 | 16.60% | | 12 |  |  | 7.11 |
|  | Apolipoprotein A-I | Q00623 | 30.615 | 16.30% | | 6 |  |  | 5.31 |
| 24 | Amine sulfotransferase | O35403 | 35.183 | 41.3% | | 201 | 3.01↓ | 0.036 | 6.08 |
|  | Nicotinate-nucleotide pyrophosphorylase [carboxylating] | Q91X91 | 31.530 | 34.8% | | 35 |  |  | 6.24 |
|  | Malate dehydrogenase, cytoplasmic | P14152 | 36.512 | 43.7% | | 29 |  |  | 6.16 |
| 25 | Not identified |  |  |  | |  | 10.94 ↑ | 0.031 |  |
| 26 | Putative hydrolase RBBP9 | O88851 | 20.912 | 19.4% | | 13 | 5.27 ↓ | 0.003 | 5.62 |

**Table 2S – List of functional networks predicted by IPA with score and number of focus molecules (analysis was performed by selecting one protein from each spot with the highest spectral count).**

| **ID** | **Molecules in Network** | **Score** | **Focus Molecules** | **Top Diseases and Functions** |
| --- | --- | --- | --- | --- |
| **1** | ACADVL,ANXA5,AQP8,ASNS,CPT2,CYP1A2,CYP2A6 (includes others),CYP39A1,DHCR24,ECI1,ELOVL2,FDFT1,FDPS,GADD45B,Gm4794/Sult3a1,Gstt3,HADHB,HSD17B10,HSD3B1,HSPA9,INSIG1,INSIG2,MBTPS1,MRPL12,NOCT,NSDHL,POR,PPARA,RORA,RORC,SCAP,SCD,Scd2,SIRT3,SLC13A1 | 7 | 5 | Lipid Metabolism, Small Molecule Biochemistry, Vitamin and Mineral Metabolism |
| **2** | ACTB,MAFB | 2 | 1 | Cellular Function and Maintenance, Cellular Growth and Proliferation, Cellular Movement |
| **3** | CASP8,CTNNB1,RGN | 2 | 1 | Cancer, Cellular Development, Organismal Injury and Abnormalities |
| **4** | GPD1,SERPINB1,SLC25A13 | 2 | 1 | Metabolic Disease, Carbohydrate Metabolism, Cardiovascular Disease |

**Table 3S - List of biological replicates**

| Sr.No. | Biological groups | Biological Replicates |
| --- | --- | --- |
| 1 | 3 months female Wild type | 4 |
| 2 | 3 months Male Wild type | 4 |
| 3 | 9 months female Wild type | 4 |
| 4 | 9 months Male Wild type | 4 |
| 5 | 14 months female Wild type | 4 |
| 6 | 14 months Male Wild type | 4 |
| 7 | 3 months female PrPC knockout | 4 |
| 8* | 3 months Male PrPC knockout* | 3 + 1 |
| 9* | 9 months female PrPC knockout* | 3 + 1 |
| 10 | 9 months Male PrPC knockout | 4 |
| 11 | 14 months female PrPC knockout | 4 |
| 12 | 14 months Male PrPC knockout | 4 |
|  | **Totol number of samples** | **48** |
| *Littermates were not available more than 3 under similar conditions, so there were only 3 biological replicates from these two groups. Therefore, extra technical replicate was used for final analysis by Delta2d DECODON software | | |
